# 2654. International Multi-Center Study Comparing the Effect of Obinutuzumab or Rituximab Treatment in Hematological Patients on COVID-19 Outcomes During the Omicron Surge

**DOI:** 10.1093/ofid/ofad500.2265

**Published:** 2023-11-27

**Authors:** Tal Shafat, Daniel Grupel, Tzvika Porges, Ana Belkin, Ofir Deri, Yonatan Oster, Shadi Zahran, Ehud Horwitz, Netanel A Horowitz, Hazim Khatib, Marjorie Batista, Anita Cortez, Tal Brosh-Nissimov, Yafit Segman, Linor Ishay, Regev Cohen, Alaa Atamna, Amy Spallone, Roy F Chemaly, Juan Carlos Ramos, Michal Chowers, Evgeny Rogozin, Noga Carmi-Oren, Şiran Keske, Orit Wolfovitz Barchad, Lior Nesher

**Affiliations:** Soroka medical center, Houston, Texas; Infectious Diseases Institute, Soroka University Medical Center, and the faculty of Health Sciences, Ben-Gurion University of the Negev, Beer-Sheva, Israel, Tel Aviv, HaMerkaz, Israel; Haematology Department, Soroka University Medical Center, and the faculty of Health Sciences, Ben-Gurion University of the Negev, Beer-Sheva, Israel, Beer Sheva, HaDarom, Israel; Internal medicine D and Infectious Diseases Unit, Sheba Medical Center, Ramat-Gan, Israel and Sackler Faculty of Medicine, Tel Aviv University, Ramat-Aviv, IsraeI, Ramat Gan, HaMerkaz, Israel; Internal medicine T, Sheba medical center, Ramat-Gan Israel, Ramat Gan, HaMerkaz, Israel; Hadassah Hebrew University Medical Center, Jerusalem, Yerushalayim, Israel; Department of Clinical Microbiology and Infectious Diseases, Hadassah Medical Center, Jerusalem, Israel and Faculty of Medicine, Hebrew University, Jerusalem Israel, Jerusalem, Yerushalayim, Israel; Department of Clinical Microbiology and Infectious Diseases, Hadassah Medical Center, Jerusalem, Israel and Faculty of Medicine, Hebrew University, Jerusalem Israel, Jerusalem, Yerushalayim, Israel; Department of Hematology and Bone Marrow Transplantation, Rambam Health Care Campus, Haifa, Israel, Haifa, Hefa, Israel; Department of Hematology and Bone Marrow Transplantation, Rambam Health Care Campus, Haifa, Israel, Haifa, Hefa, Israel; Department of Infectious Diseases, AC Camargo Cancer Center, São Paulo, SP, Brazil., São Paulo, Sao Paulo, Brazil; Department of Hematology and Cell Therapy, AC Camargo Cancer Center, São Paulo, SP, Brazil, São Paulo, Sao Paulo, Brazil; Infectious Diseases Unit, Samson Assuta Ashdod University Hospital, Ashdod, and the faculty of Health Sciences, Ben-Gurion University of the Negev, Beer-Sheva, Israel., Ashdod, HaDarom, Israel; Hematology Institute, Samson Assuta Ashdod University Hospital, Ashdod, and the faculty of Health Sciences, Ben-Gurion University of the Negev, Beer-Sheva, Israel, Ashdod, HaDarom, Israel; Rappaport Faculty of Medicine, Technion, Haifa 3109601, Israel and Hillel Yaffe Medical Center, Hadera, Israel, Hadera, HaMerkaz, Israel; Rappaport Faculty of Medicine, Technion, Haifa 3109601, Israel and Hillel Yaffe Medical Center, Hadera, Israel, Hadera, HaMerkaz, Israel; Infectious Diseases Unit, Rabin Medical Center, Beilinson Hospital, Petah Tikva, Israel, Petah Tiqva, HaMerkaz, Israel; University of Texas MD Anderson Cancer Center, Houston, Texas; MD Anderson, Houston, Texas; Infectious Disease Unit, Hospital Universitario La Paz, Madrid, Spain, Madrid, Madrid, Spain; Meir Medical Center, Tel-Aviv University, Israel, Kfar Saba, HaMerkaz, Israel; Infectious Disease unit, Shamir (Assaf Harofeh) Medical Center, Be'er Ya'akov, Israel., Be'er Ya'akov, HaMerkaz, Israel; Infectious Disease unit, Shamir (Assaf Harofeh) Medical Center, Be'er Ya'akov, Israel, Be'er Ya'akov, HaMerkaz, Israel; Department of Infectious Diseases, VKV American Hospital, Istanbul, Turkey, Istanbul, Istanbul, Turkey; Infectious Disease Unit, Shaare Zedek Medical Center, Jerusalem, Israel., Jerusalem, Yerushalayim, Israel; Soroka Medical Center, Beer Sheva, HaDarom, Israel

## Abstract

**Background:**

Hematological malignancy (HM) patients treated with anti-CD20 monoclonal antibodies are at higher risk for severe COVID-19 and adverse outcomes. A previous single-center study showed worse outcomes in patients treated with obinutuzumab than those treated with rituximab.

**Methods:**

This is an international, multi-center population-based study across 15 centers (Israel, USA, Spain, Brazil, and Turkey). We included patients with HM treated with obinutuzumab or rituximab between December 2021 and June 2022, when Omicron lineage variants were dominant.

**Results:**

We collected data on 1049 patients, of which 761 (73%) received rituximab. Of the rituximab group, 191 contracted COVID-19 compared to 104 in the obinutuzumab group (fig 1). COVID-19 patients in the obinutuzumab group were younger (mean age of 61±11.7 vs. 64 ±14.5 years, p=0.037), had more favorable HM diagnosis (aggressive lymphoma: 7.7% vs. 67.0%, p< 0.001), and were on maintenance therapy at COVID-19 diagnosis (62.4% vs. 36.2%, p< 0.001). Severe COVID-19 occurred in 31.7% (n=33) of patients in the obinutuzumab group and in 22.0% (n=42) in the rituximab group (fig 2). In a multivariable analysis for severe COVID-19, adjusted for Charlson co-morbidity index, HM status, and tixagevimab/cilgavimab (T-C) prophylaxis, we observed an odds ratio of 2.06 (95% CI 1.11-3.81, p=0.021) for obinutuzumab treatment. Prophylaxis with T-C was protective (OR 0.32 95% CI 0.10-0.99, p=0.048). In the secondary outcomes analysis, more patients with COVID-19 in the obinutuzumab were hospitalized (51.9% vs. 35.1% p=0.005), required ICU admission (13.5% vs.5.3%, p=0.014), with a non-significant difference in COVID-19 related mortality (n=11, 10.6% vs. n=12, 6.3%, p=0.189).

**Figure:**
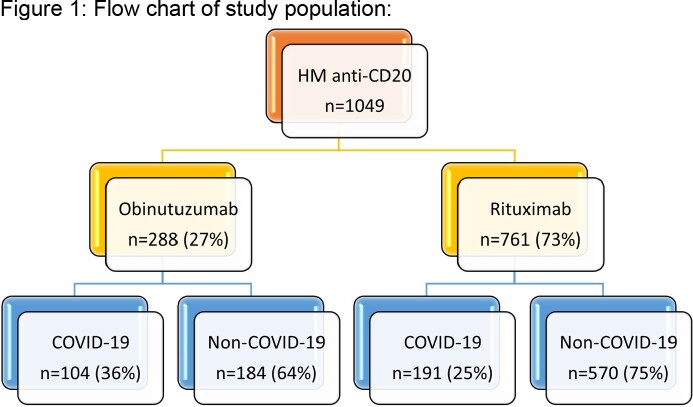

**Figure:**
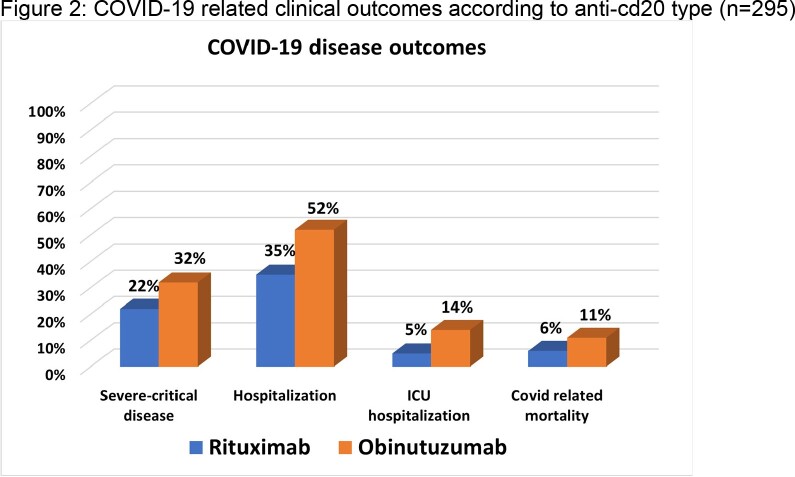

**Conclusion:**

This international, multi-center cohort study demonstrates that despite younger age and more favorable HM diagnoses, patients receiving obinutuzumab had more severe COVID-19 outcomes than those receiving rituximab. Our findings underscore the need to evaluate the risk-benefit when considering obinutuzumab therapy for HM patients and re-emphasize the crucial role of pre-exposure prophylaxis with effective anti-SARS-CoV-2 monoclonal antibodies during high transmission in the community.

**Disclosures:**

**Roy F. Chemaly, MD/MPH**, Eurofins-VViracor: Grant/Research Support|Karius: Advisor/Consultant

